# Sex and gender differences in hemostasis in critical illness

**DOI:** 10.62675/2965-2774.20260035

**Published:** 2026-03-16

**Authors:** Julie Helms, Nicole P. Juffermans, Toshiaki Iba

**Affiliations:** 1 Université de Strasbourg Faculté de Médecine Fédération Hospitalo-Universitaire TARGET Strasbourg France Faculté de Médecine, Université de Strasbourg, Fédération Hospitalo-Universitaire TARGET - Strasbourg, France.; 2 Hôpitaux Universitaires de Strasbourg Nouvel Hôpital Civil Service de Médecine Intensive-Réanimation Strasbourg France Service de Médecine Intensive-Réanimation, Nouvel Hôpital Civil, Hôpitaux Universitaires de Strasbourg - Strasbourg, France.; 3 French National Institute of Health and Medical Research Regenerative Nanomedicine Strasbourg France Regenerative Nanomedicine, French National Institute of Health and Medical Research - Strasbourg, France.; 4 Erasmus Medical Center Department of Intensive Care and Emergency Perioperative and Intensive Care Laboratory Rotterdam Netherlands Department of Intensive Care and Emergency Perioperative and Intensive Care Laboratory, Erasmus Medical Center - Rotterdam, The Netherlands.; 5 Juntendo University Faculty of Medical Science Tokyo Japan Faculty of Medical Science, Juntendo University - Tokyo, Japan.

**Keywords:** Hemostasis, Fibrinolysis, Sex, Gender, Sex factors, Intensive care units

## Abstract

Critical illness disrupts hemostasis through tightly linked inflammatory, endothelial, platelet, and coagulation pathways collectively described as immunothrombosis. These mechanisms are not sex-neutral. Biological sex and gender-related factors influence baseline coagulation profiles, vascular function, fibrinolysis, and platelet reactivity, and may therefore modify thrombotic and bleeding phenotypes observed in the intensive care unit. This short review summarizes current evidence on sex-associated differences in hemostatic biology and their clinical implications in critical illness. It outlines priorities for sex-stratified research to support more precise hemostatic management in the intensive care unit.

## INTRODUCTION

**Figure 1:**
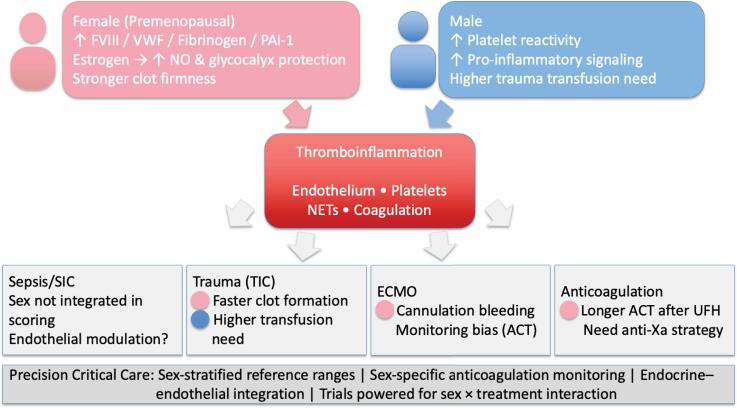
Sex and gender shape immunothrombosis in critical illness.

Critical illnesses may profoundly perturb hemostasis. The modern concept of immunothrombosis provides a framework in which innate immunity, endothelial injury, platelet activation, and coagulation cooperate to maintain microvascular integrity in the face of infection or tissue damage.^(1)^ When dysregulated, these processes drive microvascular thrombosis, consumption coagulopathy, and organ dysfunction. A key, but often overlooked, dimension is that all these pathways are not sex-neutral. Biological sex (chromosomes, gonads, hormones) and gender (roles, behaviors, care access) shape thrombotic and bleeding phenotypes before and during intensive care unit (ICU) admission (Figure 1). To date, ICU physicians routinely adjust for age, weight, renal function, and comorbidities, yet do not integrate sex despite mounting evidence that it modifies hemostatic biology.

FVIII - factor VIII; VWF - von Willebrand factor; PAI-1 - plasminogen activator inhibitor 1; NO - nitric oxide; NET - neutrophil extracellular traps; SIC - sepsis-induced coagulopathy; TIC - trauma-induced coagulopathy; ECMO - extracorporeal membrane oxygenation; ACT - activated clotting time; UFH - unfractionated heparin.

Biological sex influences baseline coagulation, endothelial function, and platelet activity. Premenopausal females typically exhibit higher levels of FVIII, von Willebrand Factor (VWF), fibrinogen, and plasminogen activator inhibitor-1 (PAI-1) with estrogen-mediated endothelial protection. In contrast, males more often display enhanced platelet reactivity and inflammatory signaling. During sepsis, trauma, or extracorporeal membrane oxygenation (ECMO), activation of the immunothrombosis axis is therefore not sex-neutral, contributing to differences in clot formation, transfusion needs, bleeding risk, and anticoagulation response. Integrating sex into hemostatic phenotyping may advance precision critical care.

### Baseline hemostatic differences relevant to intensive care unit care

Several cohorts show that females, particularly before menopause, have higher levels of factor VIII (FVIII), von Willebrand Factor (VWF), fibrinogen, and plasminogen activator inhibitor-1 (PAI-1) than males (Table 1).^(2,3)^ A recent meta-analysis confirmed that FVIII > 90th percentile confers an approximately three-fold increased venous thromboembolism (VTE) risk.^(4)^ Women also display higher C-reactive protein (CRP) and adipose-driven inflammatory signaling.^(5)^ These differences create a procoagulant baseline that can be triggered by inflammatory or endothelial stress.

**Table 1:** Baseline biological differences

Domain	Female (premenopausal)	Male
Coagulation factors	↑ FVIII, VWF, fibrinogen, PAI-1	Lower baseline levels
Endothelium	Estrogen → ↑ NO, glycocalyx protection, ↑ protein C pathway	Less endothelial protection
Platelets	Lower baseline reactivity	↑ platelet reactivity
Inflammation	↑ CRP, adipose-driven signaling	Different cytokine profile

FVIII – factor VIII; VWF - von Willebrand factor; PAI-1 - plasminogen activator inhibitor 1; NO - nitric oxide; CRP-C-reactive protein.

Studies on microvessels further demonstrate sex-specific vascular-coagulation coupling: in the Netherlands Epidemiology of Obesity cohort, impaired endothelial glycocalyx was associated with higher factor IX (FIX), FVIII, and fibrinogen levels only in women, suggesting sex-specific procoagulant vascular vulnerability.^(6)^

Hemostatic dimorphism also evolves during women's life: puberty increases gonadal hormone exposure, pregnancy induces major increases in fibrinogen, VWF, and factor X (FX), while estrogen withdrawal reduces endothelial nitric oxide and protein C pathway support. These transitions may influence who develops thrombosis or bleeding under stress, although ICU data remain scarce.

### Sepsis and septic shock

Sepsis activates the immunothrombosis axis: glycocalyx shedding, endothelial permeability, platelet-neutrophil aggregation, complement activation, coagulation activation, and fibrinolytic insufficiency. Epidemiological data suggest that men are more frequently admitted to the ICU for septic shock. In contrast, outcomes vary with age in women: some report lower mortality in older women, others show no independent association after controlling for comorbidities and frailty.^(7)^

In a sepsis cohort, women had less thrombocytopenia, lower Sequential Organ Failure Assessment (SOFA) scores, and lower crude mortality after adjustment, although sex was not independently associated with adjusted mortality.^(8)^

Mechanistically, sex hormones exert complex and sometimes opposing effects on hemostasis. Estrogens are associated with higher levels of several coagulation factors (including FVIII, VWF, and fibrinogen), supporting a procoagulant plasma profile. However, they also enhance endothelial nitric oxide bioavailability, help preserve glycocalyx integrity, and support the protein C pathway. Testosterone has been associated with increased platelet reactivity and amplification of inflammatory signaling.^(9)^ These pathways intersect with the mechanisms of sepsis-induced coagulopathy. Importantly, the clinical relevance of the more procoagulant baseline profile observed in females remains uncertain, as sepsis mortality is generally reported to be lower in females than in males in several cohorts.^(10,11)^ Yet sepsis-induced coagulopathy and disseminated intravascular coagulation scoring systems do not incorporate sex, and no anticoagulation trial in sepsis has been designed to test sex-specific treatment effects.

### Trauma-induced coagulopathy and massive transfusion

Trauma-induced coagulopathy provides another example of sex dimorphism. Premenopausal women frequently demonstrate shorter clot initiation times, stronger clot firmness, and reduced fibrinolysis after injury, consistent with higher estrogen levels.^(12)^ Clinically, this may translate into lower transfusion requirements during massive hemorrhage.^(12)^ Secondary analyses of massive transfusion studies thus show that women receive fewer units of plasma, platelets, and red blood cells to achieve similar hemostatic targets. These findings are consistent with viscoelastic studies showing more rapid clot formation and less hyperfibrinolysis in hormonally active women. When females age, however, a transition toward increased fibrinolysis compared with younger females and males has been described, again suggesting that (loss of) female hormones importantly impacts the hemostatic response to trauma.^(13)^

However, in traumatic brain injury, extracranial thrombotic and bleeding events differ modestly: men require more correction of coagulopathy. Nonetheless, algorithms to correct trauma-induced coagulopathy are non-gender specific.^(14)^

As far as ICU management is concerned, incorporating sex-specific reference ranges into hemostasis targets could further refine antifibrinolytic strategies and product ratios, particularly given that tranexamic acid (TXA) efficacy may vary with baseline fibrinolytic status.

### Intensive care unit transfusion thresholds and blood product utilization

Intensive care unit transfusion guidelines mostly rely on hemoglobin and platelet thresholds^(15)^ without differentiating by sex, despite biological differences: women have lower baseline hemoglobin, smaller circulating volume, and often demonstrate stronger clot firmness on viscoelastic testing. In trauma, some cohorts suggest lower transfusion requirements in women, although reported sex differences in transfusion requirements are inconsistent and mainly reported in non-ICU trauma cohorts.^(16)^ On the other hand, in cardiac surgery, women require significantly more red blood cell (RBC) transfusions without parallel increases in fresh frozen plasma or platelets.^(12)^

Whether ICU transfusion strategies should integrate sex is unknown, but it should probably at least be investigated in future trials.

### Extracorporeal membrane oxygenation and extracorporeal circuits

Extracorporeal circuits activate coagulation, complement, and shear-related endothelial injury, and induce acquired von Willebrand syndrome, platelet consumption, and fibrinolysis. Sex may influence thrombosis via platelet hyperreactivity, endothelial glycocalyx vulnerability, higher VWF/FVIII levels, and distinct heparin pharmacodynamics, and influence bleeding via smaller vessel caliber at cannulation, lower circulating blood volume, and relatively higher anticoagulation exposure for standard weight-based dosing.^(17–19)^ In venoarterial extracorporeal membrane oxygenation (VA-ECMO) for cardiogenic shock, women are often older at cannulation and have smaller-caliber vessels, contributing to higher cannulation-related bleeding.^(12)^ Given that activated clotting time (ACT) depends on platelet count, hematocrit, and fibrinogen (also differing with sex), anti-Xa monitoring may reduce bias, yet remains inconsistently adopted.^(20)^

Data remain scarce as no ECMO anticoagulation study has been powered to detect "sex × treatment" interactions.

### Pharmacologic anticoagulation and monitoring

Sex differences in anticoagulation response are well described outside the ICU. In a vascular surgery cohort, women developed longer ACTs after a weight-based unfractionated heparin (UFH) bolus and had higher bleeding rates despite standard dosing.^(21)^ For direct oral anticoagulants (DOACs), P-glycoprotein transport, higher adiposity, and reduced renal clearance in women can increase systemic levels. At the same time, low-molecular-weight heparin (LMWH) pharmacokinetics remain more predictable and mainly renal.^(22)^ However, robust sex-disaggregated data in ICU populations are still lacking.

## CONCLUSION

Sex and gender influence hemostatic biology, which becomes apparent in the critically ill and injured. These differences affect the expression of coagulopathies, transfusion behavior, extracorporeal thrombosis, and anticoagulation response. Although biological differences are increasingly recognized, their integration into intensive care unit practice remains limited. Priorities might include sex-stratified hemostasis reference intervals, sex-based anticoagulation strategies, and clinical trials that integrate endocrine and endothelial biology. Incorporating sex into hemostatic management may represent an important step toward precision critical care.

## Data Availability

The data cannot be made publicly available, as it is a review.

## References

[1] Engelmann B, Massberg S (2013). Thrombosis as an intravascular effector of innate immunity. Nat Rev Immunol.

[2] Conlan MG, Folsom AR, Finch A, Davis CE, Sorlie P, Marcucci G (1993). Associations of factor VIII and von Willebrand factor with age, race, sex, and risk factors for atherosclerosis. The Atherosclerosis Risk in Communities (ARIC) Study. Thromb Haemost.

[3] Lowe GD, Rumley A, Woodward M, Morrison CE, Philippou H, Lane DA (1997). Illustrative reference ranges by age, sex and hormone use. Br J Haematol.

[4] Lowe G, Wu O, van Hylckama Vlieg A, Folsom A, Rosendaal F, Woodward M (2023). Plasma levels of coagulation factors VIII and IX and risk of venous thromboembolism: systematic review and meta-analysis. Thromb Res.

[5] Karastergiou K, Smith SR, Greenberg AS, Fried SK (2012). Sex differences in human adipose tissues - the biology of pear shape. Biol Sex Differ.

[6] Yuan L, Han J, van der Velden AI, Vink H, de Mutsert R, Rosendaal FR (2023). Sex-specific association between microvascular health and coagulation parameters: the Netherlands Epidemiology of Obesity study. J Thromb Haemost.

[7] Rudd KE, Johnson SC, Agesa KM, Shackelford KA, Tsoi D, Kievlan DR (2020). Global, regional, and national sepsis incidence and mortality, 1990-2017: analysis for the Global Burden of Disease Study. Lancet.

[8] Sunden-Cullberg J, Nilsson A, Inghammar M (2020). Sex-based differences in ED management of critically ill patients with sepsis: a nationwide cohort study. Intensive Care Med.

[9] Regitz-Zagrosek V, Kararigas G (2017). Mechanistic pathways of sex differences in cardiovascular disease. Physiol Rev.

[10] Antequera A, Lopez-Alcalde J, Stallings E, Muriel A, Fernández Félix B, Del Campo R (2021). Sex as a prognostic factor for mortality in critically ill adults with sepsis: a systematic review and meta-analysis. BMJ Open.

[11] Rose N, Agrama I, Nachtigall I, Pletz MW, Rosendahl J, Chung HY (2025). Sex differences in sepsis outcomes across the lifespan: a population-based cohort study in Germany. Crit Care.

[12] Coleman JR, Gumina R, Hund T, Cohen M, Neal MD, Townsend K (2024). Sex dimorphisms in coagulation: Implications in trauma-induced coagulopathy and trauma resuscitation. Am J Hematol.

[13] Dujardin RW, Kleinveld DJ, van den Brom CE, Geeraedts LM, Beijer E, Gaarder C (2024). Older females have increased mortality after trauma as compared with younger females and males, associated with increased fibrinolysis. J Trauma Acute Care Surg.

[14] Gupte R, Brooks W, Vukas R, Pierce J, Harris J (2019). Sex Differences in traumatic brain injury: what we know and what we should know. J Neurotrauma.

[15] Vlaar AP, Dionne JC, de Bruin S, Wijnberge M, Raasveld SJ, van Baarle FE (2021). Transfusion strategies in bleeding critically ill adults: a clinical practice guideline from the European Society of Intensive Care Medicine. Intensive Care Med.

[16] Brown JB, Cohen MJ, Minei JP, Maier RV, West MA, Billiar TR (2012). Inflammation and the Host Response to Injury Investigators. Characterization of acute coagulopathy and sexual dimorphism after injury: females and coagulopathy just do not mix. J Trauma Acute Care Surg.

[17] Gasperi V, Catani MV, Savini I (2020). Platelet responses in cardiovascular disease: sex-related differences in nutritional and pharmacological interventions. Cardiovasc Ther.

[18] Lawrence JB, Leifer DW, Moura GL, Southern P, Emery JD, Bodenheimer SL (1995). Sex differences in platelet adherence to subendothelium: relationship to platelet function tests and hematologic variables. Am J Med Sci.

[19] Ranucci M, Aloisio T, Di Dedda U, Menicanti L, de Vincentiis C, Baryshnikova E, Surgical and Clinical Outcome REsearch (SCORE) group (2019). Gender-based differences in platelet function and platelet reactivity to P2Y12 inhibitors. PLoS One.

[20] Helms J, Frere C, Thiele T, Tanaka KA, Neal MD, Steiner ME (2023). Anticoagulation in adult patients supported with extracorporeal membrane oxygenation: guidance from the Scientific and Standardization Committees on Perioperative and Critical Care Haemostasis and Thrombosis of the International Society on Thrombosis and Haemostasis. J Thromb Haemost.

[21] Roosendaal LC, Wiersema AM, Smit JW, Doganer O, Blankensteijn JD, Jongkind V (2022). Editor's Choice - Sex differences in response to administration of heparin during non-cardiac arterial procedures. Eur J Vasc Endovasc Surg.

[22] Farkouh A, Riedl T, Gottardi R, Czejka M, Kautzky-Willer A (2020). Sex-related differences in pharmacokinetics and pharmacodynamics of frequently prescribed drugs: a review of the literature. Adv Ther.

